# Population Responses to Environmental Change in a Tropical Ant: The Interaction of Spatial and Temporal Dynamics

**DOI:** 10.1371/journal.pone.0097809

**Published:** 2014-05-19

**Authors:** Doug Jackson, John Vandermeer, Ivette Perfecto, Stacy M. Philpott

**Affiliations:** 1 Department of Ecology and Evolutionary Biology, University of Michigan, Ann Arbor, Michigan, United States of America; 2 School of Natural Resources and Environment, University of Michigan, Ann Arbor, Michigan, United States of America; 3 Environmental Studies Department, University of California Santa Cruz, Santa Cruz, California, United States of America; University of Sussex, United Kingdom

## Abstract

Spatial structure can have a profound, but often underappreciated, effect on the temporal dynamics of ecosystems. Here we report on a counterintuitive increase in the population of a tree-nesting ant, *Azteca sericeasur,* in response to a drastic reduction in the number of potential nesting sites. This surprising result is comprehensible when viewed in the context of the self-organized spatial dynamics of the ants and their effect on the ants’ dispersal-limited natural enemies. Approximately 30% of the trees in the study site, a coffee agroecosystem in southern Mexico, were pruned or felled over a two-year period, and yet the abundance of the ant nests more than doubled over the seven-year study. Throughout the transition, the spatial distribution of the ants maintained a power-law distribution – a signal of spatial self organization – but the local clustering of the nests was reduced post-pruning. A cellular automata model incorporating the changed spatial structure of the ants and the resulting partial escape from antagonists reproduced the observed increase in abundance, highlighting how self-organized spatial dynamics can profoundly influence the responses of ecosystems to perturbations.

## Introduction

It is evident that population dynamics occur at both temporal and spatial scales. In time, the most elementary considerations usually involve the rate of change and the final density, frequently referred to as the carrying capacity. In space, the situation is less evident, but at a minimum populations are known to occur frequently in non-random patterns. The dialectical interaction between these scales has not been a major theme in theoretical ecology, although there are obvious exceptions [1], [2]. Here we contemplate the results of a seven year study in an extended space in which we argue that it is only through the interplay of space and time that the observed dynamics of a population of ants makes sense.

**Figure 1:**
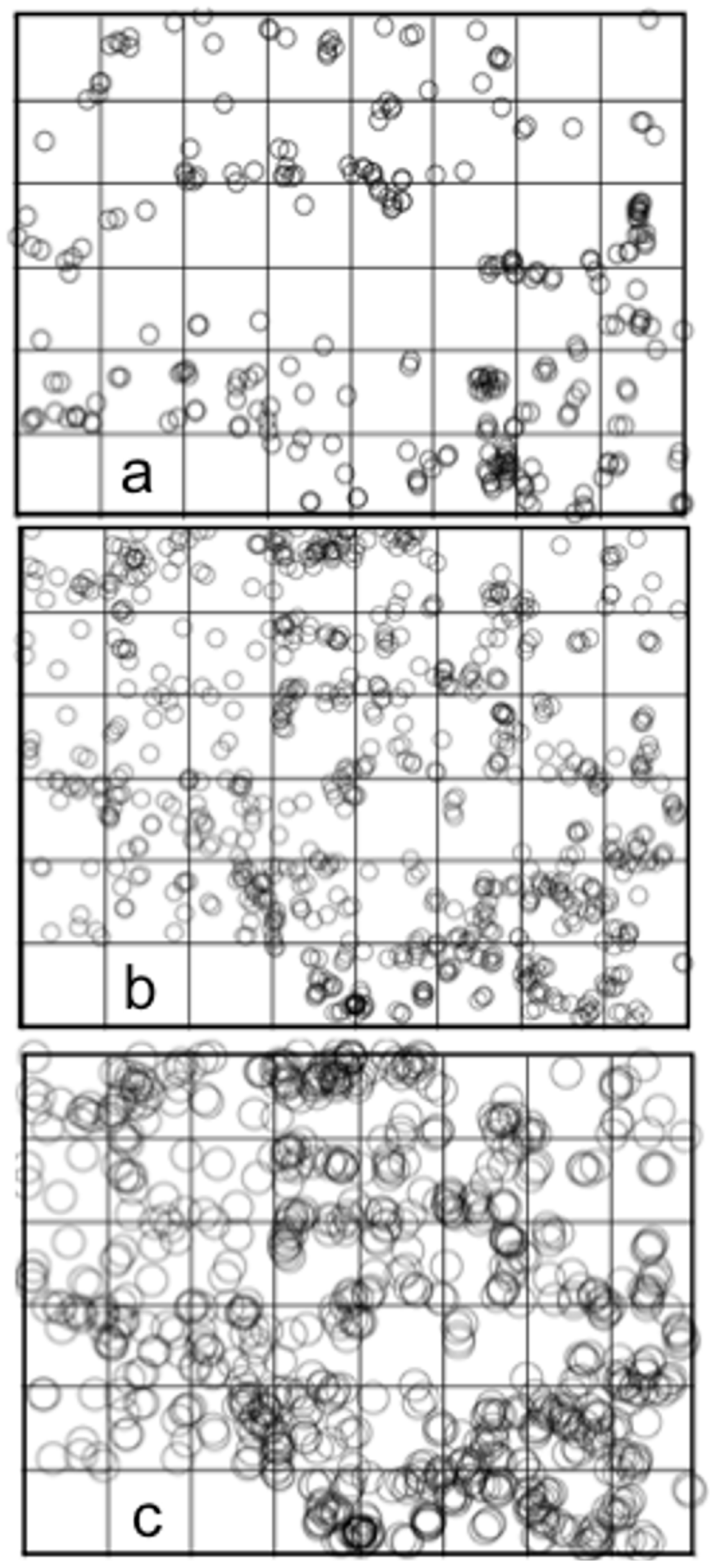
Patterns of distribution in a 45 ha permanent plot. (The three empty hectares are not sampled as part of the plot due to local geography). a. Distribution of nests of *Azteca sericeasur* in 2005. b Distribution of nests of *A. sericeasur* in 2011. c. Distribution of nests of *A. sericeasur* in 2011 at a cluster scale (diameter of the circles) of 38 meters where clusters connecting from one end of the plot to the other (a spanning cluster) are evident. Note the lack of a spanning cluster in a and b, both of which are at a cluster scale of 20 meters.

Dynamics through time are understood to be a shifting balance between the rate of population growth and the carrying capacity of the environment. At equilibrium, a population may approach that carrying capacity, or it may approach some other value dictated by other ecological connections (e.g., interspecific competitors, predators, parasites, etc.). The details are complex and specific to the particular system under consideration, but there is a general expectation that there should be a positive correlation between the attained population density and the carrying capacity of the environment. Here, we report a counterintuitive increase, over a seven year period, of the population density of a tropical, arboreal ant, *Azteca sericeasur*, in response to a drastic *reduction* of its carrying capacity (Figures 1a and 1b). This ant was previously referred to as *A. instabilis*, but has been re-identified as *A. sericeasur* due in part to the queens’ smaller ocelli and distinct yellow and brown facial markings (J. Longino, *pers. comm.).*

Dynamics in space can be framed in a parallel fashion as a balance between local dispersal and a force restricting that dispersal (parallel to birth and death), leading to a non-random spatial distribution with particular recognizable characteristics. Such a dynamic arrangement is sometimes referred to as spatial self-organization and has come to be recognized as a widespread phenomenon in nature, from the flocking of birds [3], formation of desert vegetation patterns [4], or clusters of ant nests [5]. Here we argue that it is only through an understanding of the mechanism of spatial pattern formation of the ant, *A. sericeasur*, that the paradoxical increase in its population density in response to a decrease in its carrying capacity can be fully understood.

## Materials and Methods

### Ethics and Data Availability Statements

No governmental permissions were required for work at our field site, which was a privately-owned coffee farm in Chiapas, Mexico. Future requests for permissions should be directed to Finca Irlanda, S.A.P.I. de C.V. ciruelos No. 18, fraccionamiento Los Laureles, Tapachula, Chiapas, Mexico. Field work did not involve contact with or removal of any endangered or protected species on any site. Data will be made available upon request.

### Study System

The study site is located at Finca Irlanda, a 300 ha organic coffee farm in the Soconusco region of Chiapas, Mexico (15° 11′ N, 92° 20′ W). The farm receives ca. 4500 mm of rain annually and is located between 900–1150 m elevation. According to a standard classification, the farm is a commercial polyculture, with almost 100 tree species in total, largely dominated by *Inga* spp.

**Figure 2:**
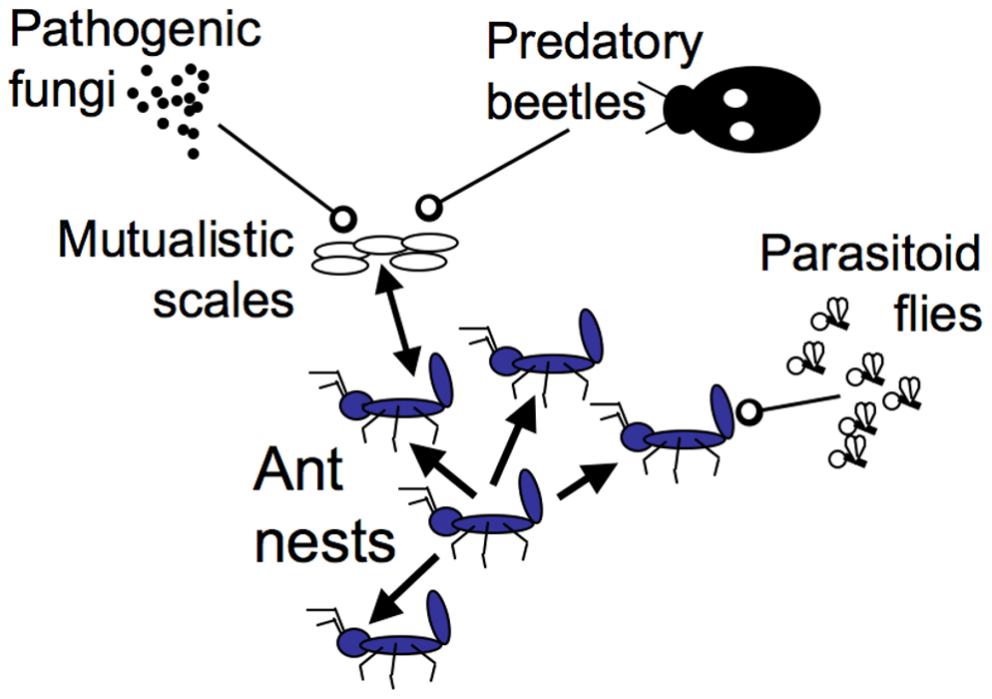
Cartoon version of the local dynamic forces. Single-headed arrows depict the satellite expansion of a cluster of ant nests. Double-headed arrows depict the mutualism between the ants *(Azteca sericeasur)* and the scale insects *(Coccus viridis).* Arrows with open circles indicate the antagonistic effects of the parasitoid phorid flies on *A. sericeasur;* and of the pathogenic fungus *(Lecanicillium lecanii)* and the predatory beetle *(Azya orbigera)* on the scale insects.

The overall community dynamics of the system in general have been extensively reported [5]–[14]. In the system of concern there exists a complicated ecological network responsible for autonomous biological control of several coffee pests [10], [14]. Control emerges from a keystone species, the tree-nesting ant *A. sericeasur,* which is dynamically connected to several predators, parasites and pathogens (Figure 2), all of which together are reported to regulate the pest assemblage [14]. Key to this system is the way in which its dynamics emerge in a spatial context [5], [7], [8].

Spatial structure in this system is determined primarily by the distribution of *A. sericeasur* colonies, which nest in the shade trees and only rarely nest in the coffee bushes themselves. The distribution of shade trees is essentially uniform, with the exception of minor clustering around roadways, but the ant nests are significantly clustered [5]. Colony formation and death have been observed to occur relatively rapidly in this system, with the population increasing as much as 29.6% in a single year (2007–2008) during the study period. This is due to the ability of *A. sericeasur* to form new nests via colony fission, or budding, in which individuals from an existing colony split off to form a new colony [15], resulting in a highly dynamic process of expansion, splitting, and disappearance of nest clusters [5].

To explore these dynamics, a 600×800 m (48 hectare) plot was established within which all shade trees were located and mapped to the nearest 2 m (usually to the nearest 1 m, but at times the terrain became too difficult to maneuver and we relaxed the precision in those areas). Only 45 of the 48 hectares were included since three of the hectares in one corner were located next to a cliff that was inaccessible due to the steepness of the terrain. At the time of mapping, each tree was affixed with a numbered aluminum tag; hammering nails to attach tags invariably aroused a swarm of ants if the tree was occupied by an *A. sericeasur* colony. Thus the mapping of trees and ant colonies was done simultaneously during the first census, in 2004. Subsequent annual censuses were done by locating every shade tree, mapping the new recruits, and determining whether or not they contained a nest.

The dependence of the ants on shade trees for nesting sites; the fact that only a single colony can occupy a shade tree at a given time; and the ease with which the presence or absence of a colony in a particular shade tree can be ascertained provide both a clear measure of the carrying capacity of the environment and a precise measure of how the population responds to changes in the carrying capacity. Unlike many organisms, for which the individual is the natural unit of abundance, for eusocial organisms such as ants the colony is a more natural unit of organization [16]. Although the abundance of colonies does not necessarily correspond to the total biomass, it does reflect the number of “individuals” that are currently occupying discrete sites in the available habitat.

The *A. sericeasur* ants tend scale insects (primarily *Coccus viridis)* on nearby coffee bushes, and harvest a sugar-rich honeydew excreted by the scales. The ants protect the scales by vigorously harassing natural enemies: parasitic wasps (mainly two species of Encyrtidae) and a coccinelid beetle (*Azya orbigera*) routinely attack the scales (Figure 2), and reduce their local population density to almost zero in the absence of ants [17]. The coccinelid beetle is myrmecophilous and thus extremely difficult to encounter except in the vicinity of the *A. sericeasur* colonies [9], [18].

The scale insects, which are mutualists of the ant *A. sericeasur,* are themselves attacked by a fungal pathogen, *Lecanicillium lecanii* [7], [19], which evidently responds to the spatial pattern of both the ants and scale insects [14]. It is clear that both the coccinelid beetle and the fungus have a potentially negative indirect effect on the ant population, through their effective attack on the scale insect (Figure 2).

A final element of importance to the present study is a group of parasitic flies (Phoridae) that exhibit density-dependent foraging (dependent on the local density of the ants) [10], [12]. The abundance of the phorid flies (and of the predatory beetle *A. orbigera*) has been found to significantly increase with an increasing density of ant nests (Hsun-Yi Hsieh, personal communication). It was originally reported [5] that these flies provided the spatially-specific negative force that was part of the spatial pattern formation, i.e., trees containing ant nests are clustered, with a power function (a straight line on a log-log plot of the frequency of clusters versus cluster size) closely fitting the distribution of clusters, despite the statistically uniform background distribution of shade trees. As detailed elsewhere [5], it appears to be the local establishment of satellite nests of *A. sericeasur* in neighboring trees, coupled with a predatory (or parasitoid) control acting preferentially on locally dense concentrations of ant nests, that gives rise to a self-organized spatial pattern of nests. These observed dynamics are illustrated in Figure 2.

In 2007 and 2008, the managers of the farm carried out a program of drastically reducing the shade cover in the farm by felling a large fraction of the shade trees. Pruning, and at times felling, shade trees is a normal part of managing a shade coffee plantation, and is required to maintain the shade cover within a desired range. However, the cuttings in 2007 and 2008 were intended to significantly lower the shade level below the previous management targets, and were therefore much more severe than normal. In the 45 ha plot, more than 30% of the original 12,227 shade trees were cut over this two-year period.

### Measurement of Spatial Pattern

An important determinant of the susceptibility of the ant population to a dispersal-limited pathogen, predator, or parasitoid is the distance to nearest neighboring nests. Thus we calculated Ripley’s K, a measure of spatial clustering [20], to estimate this potential. To calculate Ripley’s K, the number of other nests in the neighborhood of each nest is compared with the number expected for a random (Poisson) distribution. The neighborhood is defined by a sampling circle with a specified radius. To determine the degree of spatial clustering at different spatial scales, Ripley’s K is calculated for a range of sampling circles. Deviations from the random expectation indicate that the spatial pattern is either more clustered or more uniform than random, depending on the direction of the deviation.

As mentioned earlier, previous work had established that the distribution of clusters of nests was well approximated by a power function for the first two years of the study [5]. As has been noted by many authors, such a pattern is precisely what would be expected when the system is self-organized, i.e., with large-scale spatial patterns arising through local-scale interactions [21], [22]. However, even if self-organization were not the underlying rule, the slope and intercept of a power function provide a statistical summary of the cluster size distribution. In all cases reported here, the distribution of the cluster sizes did indeed fit a power function well, and we take the power function parameter estimate (the slope) as a measure of the spatial patterning of the system (e.g., in a perfect power function fit the power function parameter is equal to the frequency of the smallest clusters divided by the size of the largest cluster – an intuitive way of grasping with a single number the overall spatial distribution).

Although cluster size distributions have been discussed copiously in the literature [22]–[26], in the particular case of point distributions in space there is a biological contingency that emerges that has not previously been dealt with, but which is crucial to understanding our results. Precisely what constitutes a cluster of nests depends on the scale of influence surrounding the nest itself, what we refer to as the cluster scale. For example, using the data from 2011, cluster scales of 20 m and 38 m are illustrated in Figure 1b and Figure 1c respectively. Note that at a scale of 20 m, there is no single cluster that spans the entire plot, but at 38 m there is one giant cluster that spans top to bottom and left to right. The cluster size distribution will depend on the scale that is chosen to decide which nests belong to the same clusters (compare Figure 1b with Figure 1c: the cluster scale is the diameter of the circle surrounding each nest). Consequently, we adjusted the cluster pattern with the cluster scale, examining all cluster scales from 20 m to 70 m, and fitting a power function to each data set (all scales, at 1 meter intervals, for each of the seven years of the study). Our general expectation was that because of the dramatic change caused by the change in management, we would see a change in the slope and the intercept of the power function reflecting changes in the distribution of ant nests.

### Computer Model

The basic mechanisms underlying the self organization process – satellite expansion of ant nest clusters and density-dependent mortality of colonies – were previously encapsulated in a discrete-time cellular automata model that successfully reproduced the observed spatial distribution of nests [5]. The shade trees were represented as a two-dimensional lattice of cells, with each cell being either empty or occupied by an ant nest at a given time, *t.* The probability of an empty site becoming occupied in the next time step, *Pe,* was an increasing function of the density of nests in the surrounding 8-cell neighborhood (known as the Moore neighborhood) in time *t:*

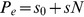
(1)where *N* is the number of neighboring cells (shade trees) occupied. The states of all cells at time *t*+1 were thus a function of the state of the system at time *t*, known as synchronous updating. Mortality was presumed to be a consequence of a phorid fly parasitoid acting in a density-dependent manner and given as:



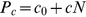
(2)The parameters of this model are the two density-independent terms (*s_o_* and *c_o_*) and the two density-dependent terms (*s* and *c*). All four parameters were estimated using data from the recurring surveys. The model generated remarkably good correspondence with the population density and general spatial pattern of the ant nests prior to the major cutting of shade trees in 2007 and 2008 [5].

A naive modification of the model to reflect the cutting of the shade trees would be to simply eliminate 30% of the trees (sites) in the model by marking them unoccupiable. Implementing the model in this way caused the extinction of the system, clearly calling into question the model (since the population in nature did not collapse). From a biological perspective, this implementation implies that during the satellite expansion of ant nests, which occurs when an existing colony buds off and occupies a neighboring, unoccupied shade tree, the ants only consider trees within a strict search radius. If there are no free shade trees in this search radius, it is assumed that the ants do not establish a new colony.

An alternative and potentially more biologically realistic assumption would be that an expanding colony will preferentially establish a satellite nest in the closest available shade trees, but will search in a wider radius if no free sites are found nearby. To approximate this behavior, we modified the satellite expansion of the original model in two ways. First, for the expansion probability, *P_e_,* the relevant neighborhood was expanded to be the Moore neighborhood plus the next nearest 16 cells, i.e., the 5-by-5 square of cells centered on the focal cell. Second, rather than populate the central unoccupied cell with a nest based on the calculated local density-dependent probability (i.e., equation 1), each occupied central cell produced a bud that occupied one of the 24 adjacent cells in the expanded neighborhood (first examining the inner square of 8 cells, then the next square of 16 cells if no available site was found in the inner square). If more than one position was available in the inner circle (or in the outer circle if no available cell was encountered in the inner one), occupancy was determined randomly.

The relevant neighborhood in the density-dependent mortality function, *N* in equation 2, was also expanded to include the 16 cells surrounding the Moore neighborhood. Although the behavior of the purported natural enemy was not changed, the calculation of local population density of the ant implies that the natural enemy is searching a wider area than before, and thus has an elevated searching horizon, either because the behavior of the natural enemy changed or the actual species of natural enemy changed.

As with the original CA model, the study system was represented by a 120×90 grid of cells on a torus to eliminate edge effects (i.e., cells on the left and right edges of the space were treated as neighbors, as were cells on the top and bottom edges). At each time step, the probabilities described above were calculated for each cell based on the distribution of nests in the previous time step, and a pseudorandom number was drawn from a uniform distribution to determine if the cell would become occupied or empty. The model was initialized with a random distribution of occupied cells and then run for a sufficient number of steps to reach a stationary distribution.

## Results

**Figure 3:**
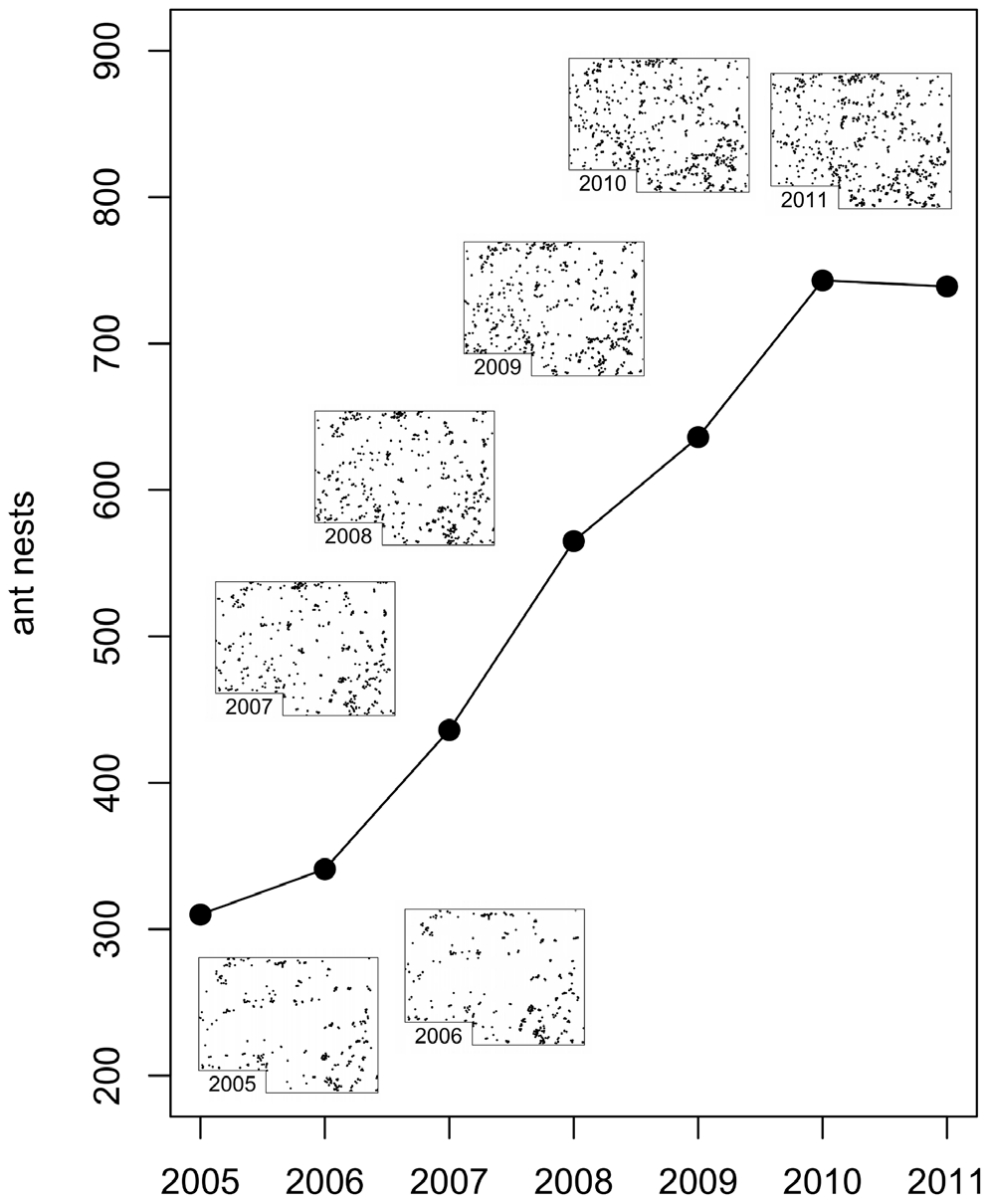
Ant nest abundance and spatial plots of ant nest distributions for all survey years.

Counter to what we expected, and counter to what a naive modification of the original CA model would predict following the removal of 30% of the shade trees, the drastic reduction in available nesting sites beginning in 2007 was associated with a substantial increase in the abundance of *A. sericeasur* colonies in the field (Figure 3). From 2005 to 2007, the number of nests increased from 310 to 436, a 41% increase. Following the second pruning in 2008, and continuing through 2010, abundance rose to approximately 750 nests, a 142% increase from the initial census; between 2010 and 2011, the number of nests remained relatively constant at this higher value. In marked contrast, a naive modification of the original CA model, i.e., simply removing 30% of the shade trees at random, predicts a drastic decline and extinction of the ant population within a few years post-pruning.

**Figure 4:**
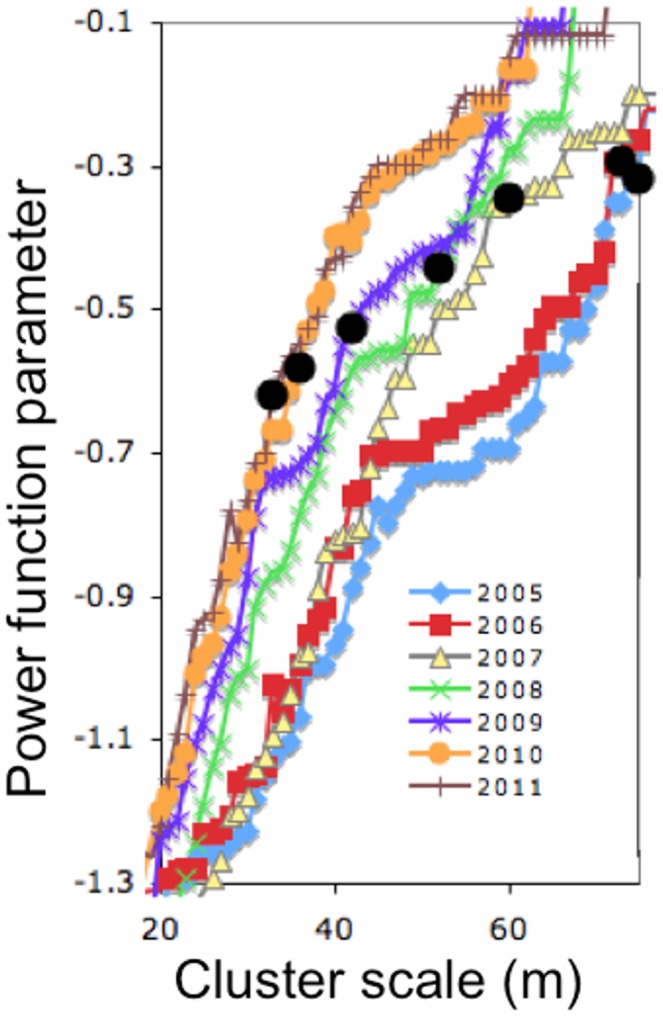
The power function exponent at all cluster scales from 20–70 m, for the seven years of the study. Note years 2005/2006 show similar pattern as do 2010/2011, whereas intermediate years show intermediate pattern. Large filled black circles are power function exponent at precisely the cluster scale that yields the first spanning cluster (i.e., the minimum cluster scale for which a spanning cluster emerges).

The spatial pattern was modified substantially, but always retained a clearly clumped pattern (Figure 3). Exploring the changes in cluster size distribution during the transition, we present the power function parameter (the exponent of a power function fit to the distribution of nest cluster sizes, or the slope of the power function on a log-log plot) for all relevant cluster scales for all seven years of the study (Figure 4). The pattern for years 2008 and 2009 falls between the patterns for years 2005/2006 on the one hand and 2010/2011 on the other, precisely what would be expected if the patterns changed as a consequence of extensive pruning and cutting. The first survey that was performed after completion of the first round of cutting, in 2007, falls intermediate, following a pattern similar to 2005/2006 at low cluster scales but moving more towards the pattern of 2008/2009 at higher cluster scales. Furthermore, the pattern is consistent in having an increasing power function parameter over time, at any fixed cluster scale. What is clear is that, much as in the case of population density generally (Figure 3), the spatial pattern underwent a systematic transformation (Figure 4).

The general spatial trend also follows a specific pattern with respect to “criticality.” Although the literature on criticality is large and itself quite complex [21], [27], a fundamental idea is that there is a critical point in clustered spatial distributions when a single cluster extends from one end of the lattice to the other, in other words, when there is a spanning or percolating cluster [28], [29]. Therefore it is instructive to examine the point of criticality for each year of the study to see if a consistent pattern of change occurs. Thus, in Figure 4 we also plot the critical points for each year (the larger solid black circles). In the years before the cutting (2005/2006 the critical power function exponent was about −0.3 and at the end of the study (2010/2011) it stands at −0.6, effectively double in absolute value. The intermediate years go from −0.35 to −0.44 to −0.53, seemingly making a smooth transfer to a new state. Perhaps more importantly from a biological perspective, the critical cluster scales (the smallest scales that generate a spanning cluster) also systematically change from about 74 m in the pre cutting years to about 35 m presently, with intermediate changes going from 60 m to 52 m to 42 m.

**Figure 5:**
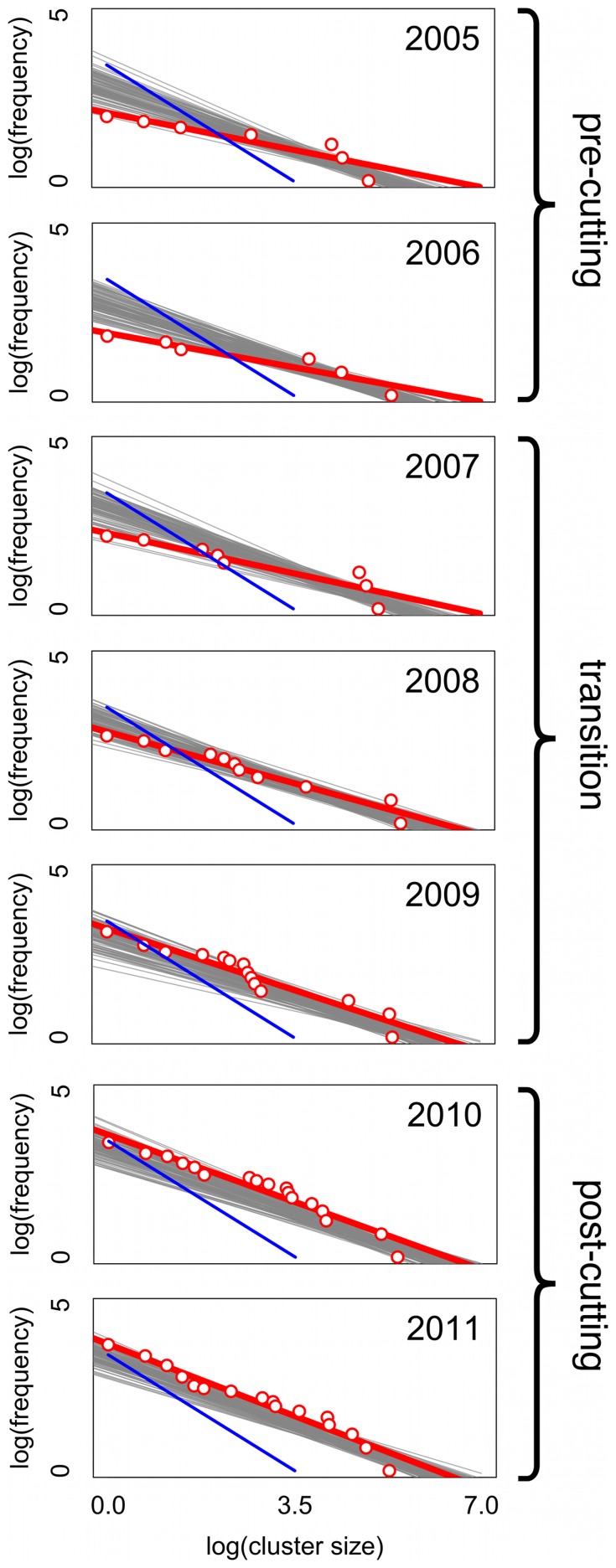
Fits (red lines) to a power function for the data (red circles) from 2005–2011, along with 100 random trials (gray lines) for each date. Random trials are based on the nest densities in each year and appear to be consistent for all years. Cluster scales in all cases are based on the spanning cluster scale (see supplementary material). Blue lines (power function exponent = −1.0, with intercept fixed at 2011 level) are constant over all panels, and are included to facilitate comparison across panels.

To observe the difference between the observed patterns and a random expectation, we generated 100 artificial nest distributions based on the nest densities in each year. After calculating the critical cluster scale for each year (based on random positioning), we computed best-fit power functions to each of the artificial distributions. Those power functions, along with the field data are presented in Figure 5 (note, all power function fits are based on the critical cluster scale, i.e., the smallest scale that gives a spanning cluster, the black symbols in Figure 4). It is evident that the states in 2005–2006 and 2010–2011 are distinct from one another and the three year transition period moves rather smoothly from one to the other, both in the parameter of the power function (slopes of the lines) and the position of the power function relative to the random expectation.

**Figure 6:**
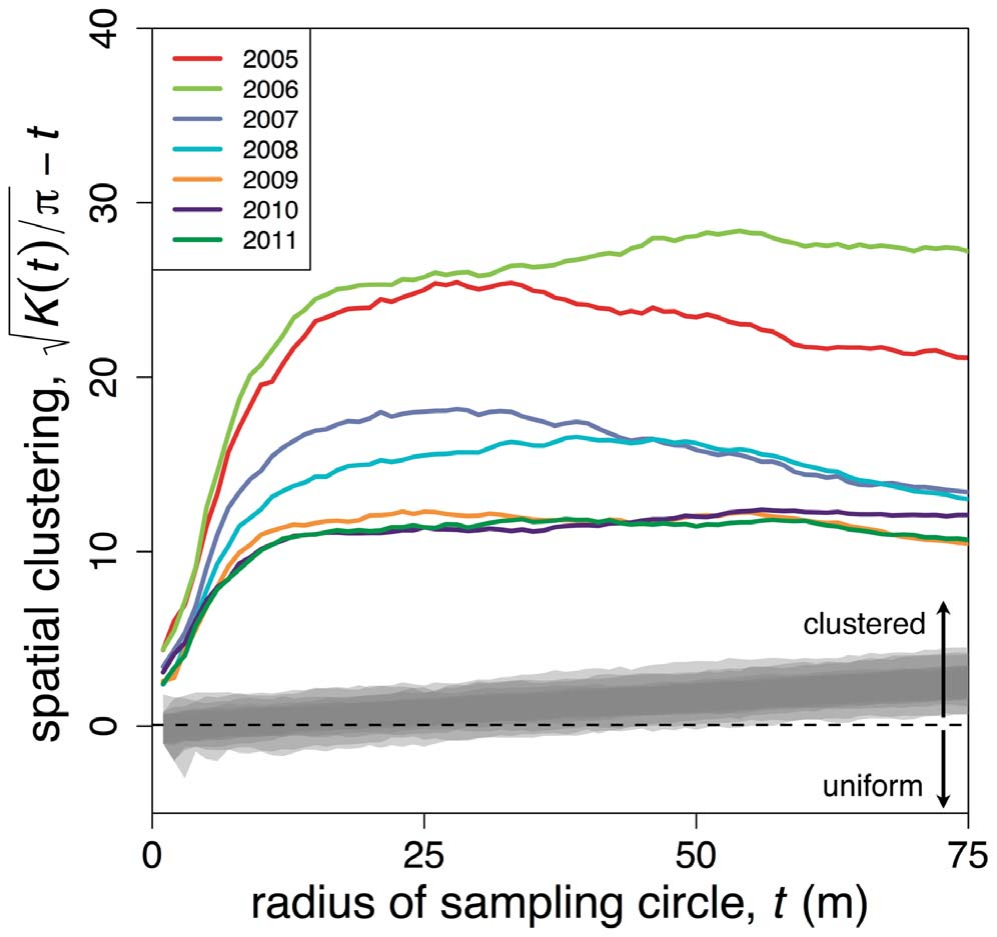
Ripley’s K for all the years, along with 1000 random allocations (in grey). Ripley’s K is a measure of clustering across a range of spatial scales, transformed here such that values above zero represent clustered distributions and values below zero are uniform distributions [20]. The ordinate effectively represents the average number of nests found within a random circle of the radius on the abscissa. The slightly positive slope of the random allocations is a consequence of the three empty hectares at the corner of the plot.

Note (Figure 4) that the critical cluster scale that leads to a spanning cluster declines over the course of the study (from about 70 m in 2004–2005 to about 30 m in 2010–2011), clearly a consequence of the increasing population density. But from the point of view of a potential natural enemy of the ant, the important question is a local one, not the question of moving from one end of the large plot to the other (what would be implied by the black circles in Figure 4) but rather the question of moving from one nest to the nearest adjacent nest. To probe this question, we calculated Ripley’s K, a common measure of clustering [20]. As shown by Ripley’s K (Figure 6), the ant nests were significantly clustered at all spatial scales between 0 and 75 m for all of the survey years. The degree of spatial clustering, however, was substantially decreased between the pre-pruning years (2005–2006) and the post-pruning years, with the transitional years lying intermediate on the spatial clustering scale. Any organism interacting in a density-dependent fashion would face a distinctly different local situation after the transition compared to before the transition, which is to say, on average it would be a longer distance between any nest and its nearest neighbor in the later years, even though the overall density of nests over the entire plot had increased.

**Figure 7:**
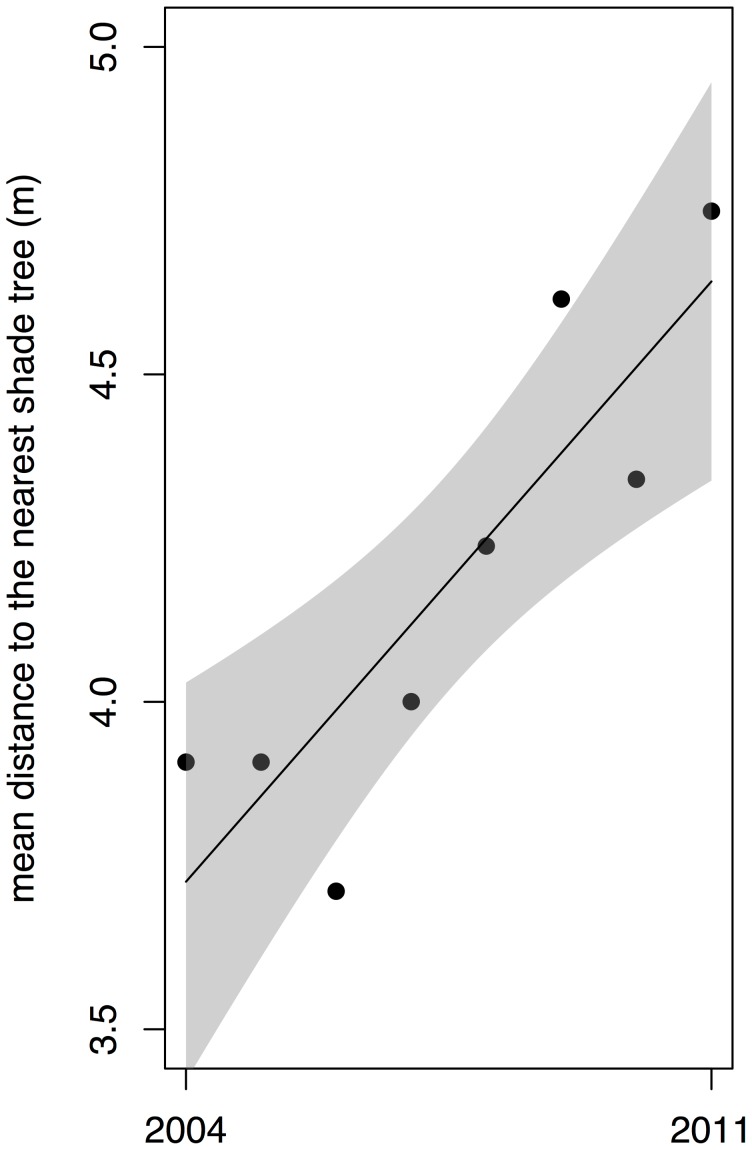
Mean distance from ant nests to the nearest shade tree. Shaded region shows 95% confidence intervals. *R^2^* = 0.72, *P*<0.01.

The pruning also substantially altered the local density of nest sites available to the ants, a key factor in the satellite expansion process that counterbalances the effect of density-dependent mortality. The mean distance from the ant nests to the nearest shade trees increased significantly during the study period (Figure 7). Considering the number of shade trees available within 9.5 m of a nest, approximately equivalent to the Moore neighborhood in the CA model, or 18.9 m, roughly equivalent to the Moore neighborhood plus the next-nearest 16 cells, the local density of available nest sites was also seen to decline from 2004 to 2011 (Figure S1). For all of these measures of nest site availability, there is some suggestion of an initial stationary period, a transitionary period following the pruning, and a new equilibrium in the post-pruning years.

**Figure 8:**
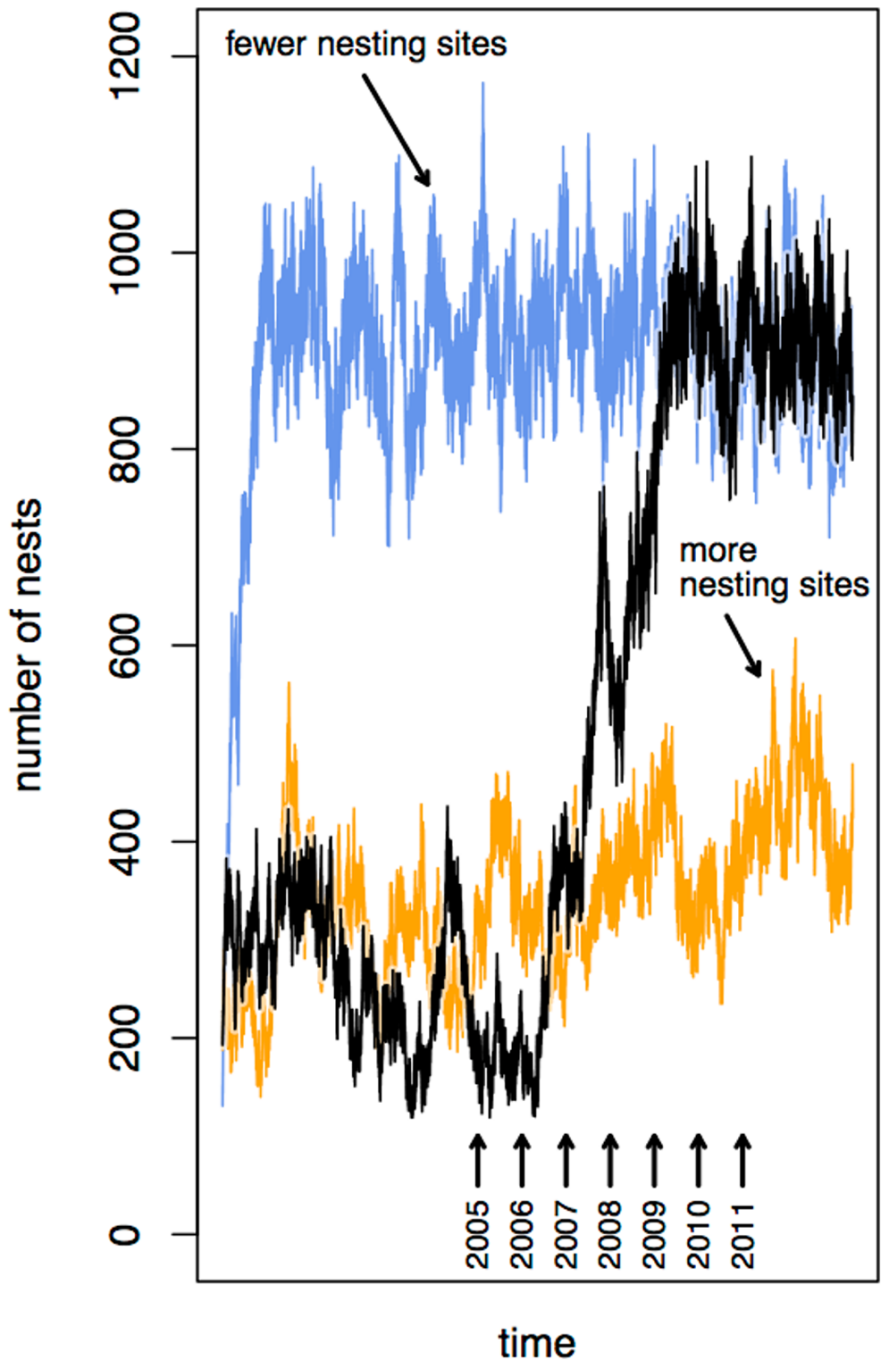
Results from modified cellular automata model [5]. The original model was modified to allow for further expansion of nests in space. Lower orange run is with original, larger, carrying capacity. Upper blue run is with modified, lower, carrying capacity. Intermediate black run begins with the larger carrying capacity, with 30% of the trees eliminated halfway through the run. Positions of years are approximate representations of the correspondence of model output with the qualitative nature of the field results.

The CA model, modified to account for the expansion of the ants’ search radius in response to a lack of available shade trees in the immediate vicinity of the parent nest, successfully captured the qualitative response of the ant population to the reduction in nesting sites. With the original number of nesting sites, the model reaches an equilibrium of approximately 400 nests (Figure 8). If the modified model is initialized with 30% of the shade trees removed at random, the equilibrium population is approximately doubled as a consequence of the decrease in the density of the ant nests, which provides a reduction in the density-dependent mortality effect of the ants’ exploiters (the phorid fly, *L. lecanii*, or another dispersal-limited enemy). When the random pruning is applied after the model reaches equilibrium, we see a multi-year transition period followed by variation around the new, higher equilibrium, as was observed in the field surveys (Figure 3).

## Discussion

Despite what intuition might have led one to expect, and counter to the predictions of the original cellular automata model of the system, a drastic decrease (approximately 30%) in the carrying capacity led to more than a doubling of the abundance of *A. sericeasur* colonies. We know of no other example in the literature that is as straightforward as this one, where carrying capacity is so clearly defined, where it clearly decreased substantially, and where the population responded by dramatically increasing. The question of how this happened is thus of interest. To fully appreciate what may have happened, we argue that the spatial distribution of the population is of importance. Such a counterintuitive result is perplexing when considered from the perspective of classical theory of population dynamics, but becomes comprehensible when viewed as a dynamic response of a self-organized system.

The population in question has been cited frequently [5], [7], [14] as having a strikingly non-random spatial pattern. The nests are clearly clustered in space, and the pattern of those clusters is well-approximated by a power function [5]. Such patterns are commonly associated with self-organization, which in this case involves a “birth rate” (probably mostly a budding rate in which parts of nests are budded off and moved to nearby trees) and a death rate, both responding to a local scale, resulting in a spatial pattern at a larger scale.

The maintenance of the power law distribution of cluster sizes – considered a signal of self organization [22] – suggests that the spatial structure of the system continued to be strongly shaped by endogenous drivers throughout the transition between the low-density, pre-pruned state and the higher-abundance, post-pruning condition. The self-organization mechanisms at play in this coffee agroecosystem appear to have remained broadly consistent throughout the study period: satellite expansion of the ant colonies tends to cause expansion of clusters, but this tendency is counteracted by the density-dependent mortality inflicted by their dispersal-limited enemies. However, the details of the spatial structuring changed in ways that seem to have fundamentally altered the key mechanisms that underlie the self organization process.

First, from the perspective of the ants, the local density of available nesting sites, whether measured in terms of the mean distance to the nearest shade tree or the mean number of shade trees in the vicinities of existing ant nests, was significantly reduced by the pruning. The relative paucity of local nesting sites appears to have forced the ants to increase their local search radius when establishing new nest sites, thereby decreasing the degree of clustering of the nests, as reflected by Ripley’s K. Consequently, the spatial structure from the perspective of the controlling agent (or agents) changed as well, with the decrease in the local density of ants causing a reduction in the density-dependent mortality effect that serves to counteract the satellite expansion of the nest clusters.

The overall reduction in the local density of nests occurred despite the increase in the abundance of nests observed during the study period. An increase in dispersal distance and an increase in abundance have countervailing effects on the compactness of clusters (Figure S2). In the current study, the density-decreasing effects of longer dispersal distances more than balanced the density-increasing effects of more ant colonies.

In addition, however, the clustering scale at which a single cluster of nests could span the study site, i.e., the percolation threshold, decreased throughout the course of the study. This metric gives some indication of how far a dispersal-limited exploiter of the *A. sericeasur* colonies would have to travel between nests in order to access the entire population of ants. The net effect of these two changes, one positive from the perspective of a controlling agent (the decrease in the cluster scale) and one negative (the decrease in the local clustering of nests), is unclear at present; it is plausible, though, that these changes caused a shift in the relative importance of the putative controls.

By forcing the ants to search further afield for nesting sites, it seems that the pruning changed the background conditions in which the potential natural enemies must search, and indeed may have transformed the system by changing the particular natural enemy that is most effective. It could have been the case, as originally suggested [5] that the phorid fly parasitoid was the main agent of control while under the new system either the beetle [9], [18] or the fungal disease [7], [19] may be the culprit. Current evidence is insufficient to favor one or the other, but the change in the nature of the spatial distribution suggests this further mechanistic question. From within the confines of a cluster of nests in 2005, a natural enemy would likely encounter another nest within 5–10 meters, while in 2011 that distance would be more like 10–15 meters.

There are, as is almost always the case, alternative mechanisms that could have produced the results described here. We have proposed that the combination of the change in population density coupled with the systematic changes in the spatial pattern suggest a shift in the way the natural enemies in the system function. However, since this was a “natural” experiment, we have no control plot and our detailed field surveys of natural enemies were restricted by insufficient resources, so we are unable to say whether or not there was an observable change in any of them, or, for that matter, whether perhaps an additional natural enemy may have emerged as a consequence of the shift. Thus we have been forced into a scientific mode, not unusual for sciences in which experimentation is difficult (e.g., astronomy), of developing mechanistic models and searching for relevant patterns that might fit with those models and shed light on the original observations. We find that the systematic changes in both temporal and spatial dynamics are reflected in our basic model when our mechanistic interpretation of the change in local dynamics is incorporated (i.e., ants searching more broadly and creating less compact local clusters of nests, with a consequent change in the local dynamics of natural enemies).

It is worth noting also that the shift in management also occurred as climate change is reportedly altering background conditions. In the case of the southern mountains of Mexico, this change is basically a small elevation in temperature and a seemingly less predictable rainfall pattern. Clearly, such changes are correlated with the deforestation of the farm, and we have no way of disentangling these two sources of variation. However, searching for nests of *A. sericeasur* at farms lower in altitude does not give us the impression that nest densities are correlated with temperature, since farms at lower altitudes (with higher temperatures) seem to have nest densities more similar to the densities at our study site in 2005, when temperatures were lower than at present.

The phenomenon documented here may apply to any victim-exploiter system with dispersal-limited exploiters, dispersal-limited victims, and establishment of the victim subject to the presence of a third factor, whether biotic or abiotic. For example, in the classic Janzen-Connell formulation [30], [31], dispersal of tree seeds is highest near to the parent tree, while the abundance of pathogens and/or predators also increases with proximity to adult trees, resulting in maximal recruitment at an intermediate distance from the parent. Many tropical trees rely on animals for seed dispersal, and the behavior of these animal dispersers may be contingent on the presence of suitable habitat. For instance, the seeds of the tropical rainforest tree *Dipteryx oleifera* are dispersed by frugivorous bats, which eat the fruits, fly to perching sites in the leaves of bat roosting palms, and then defecate the seeds, creating seed piles from which the majority of seedlings emerge [32]. One can imagine that a drastic reduction in the number of the palms, which would seem at first to be detrimental to the trees (bats roost in the palms, and *D. oleifera* depends on bats for dispersal, so fewer palms should be bad for the trees), could actually cause an increase in tree abundance through the indirect effects on the host-predator dynamics between *D. oleifera* and its primary predators, which are insect larvae.

In light of the potential for the sort of complex, possibly counterintuitive responses that we have documented here, it is important to consider the possibility that further dynamic shifts could continue to transpire – that an equilibrium may never be reached. As but one example in many that could be imagined, if an increase in the dispersal distance of the victim (*A. sericeasur* in our system) were to shift the competitive landscape of the exploiters, thereby advantaging a particular exploiter with a longer dispersal distance, that exploiter’s population may increase, perhaps driving the victim’s population down again in the medium term. Non-numerical responses of both the victims and exploiters to the altered spatial structure, e.g., due to behavioral plasticity, could also cause further spatial and temporal dynamics. More complete knowledge of the dispersal distances, dynamical characteristics, and possibly the evolutionary characteristics of the relevant organisms, combined with an understanding of the mechanisms underlying the generation of spatial structure in the system, would be essential for predicting the response over the medium and long term.

It is also worth noting that *A. sericeasur* is at the center of a complex system that is involved with the “autonomous” control of three of the major pest organisms of coffee [6], [14], [33] and is thus of potentially large practical importance. *Azteca sericeasur* is one of those organisms that, after a cursory examination, seems to be a pest itself, due to its mutualistic association with the scale insect [13] which itself is a potential pest. Yet there is now substantial evidence that the ant is a direct predator on the coffee berry borer (*Hypothenemus hampei*) [34]–[37]. The scale insects are held under control by a beetle (*A. orbigera*), whose larvae are protected from the ants and thus permitted to prey on the scales locally, but whose adults fly afar, locating scale insects well-removed from protection from the ants [8], [9], [18]. Furthermore, the most devastating disease of coffee, the coffee rust disease caused by the fungus *Hemileia vastatrix*, is kept partially under control by the pathogenic fungus *L. lecanii* [6], [7], [38], which is also pathogenic on the scale insects; *L. lecanii* only reaches epizootic levels when the scales are locally dense, which only happens when the ants are tending them. This suite of species and interactions functions, we have argued extensively, to maintain these three coffee pests at acceptable levels within the system. In this context, understanding the cause of dramatic changes in the distribution of the keystone species of the system seems quite important.

In light of the central role that self-organization can play in the spatial structuring of ecosystems [39], [40] and the potential for counterintuitive responses of ecosystems so structured to perturbations (as shown in the present study), coupled with the accelerating pace, scale, and intensity of ecosystem alteration, the need for increased understanding of the phenomenon of self-organization is clear. As shown by the striking results documented here, an intuition informed by interactions with non-self-organizing systems may lead to predictions that are diametrically opposed to what might actually transpire in the event of a change in the environment or a modification of management practices. In the current age of major anthropogenic habitat destruction and ecosystem modification, educating our intuition to reflect the nonlinear behavior of complex, spatially structured systems is essential.

## Supporting Information

Figure S1 **Mean number of shade trees adjacent to ant nests.** Dashed line (*R^2^* = 0.78, *P*<0.01) shows the mean number of shade trees within 9.5 m of ant nests, which corresponds to the eight nearest cells, i.e., the Moore neighborhood, in the cellular automata model. Solid line (*R^2^* = 0.80, *P*<0.01) shows the mean number of shade trees within 18.9 m of the ant nests, which corresponds to the Moore neighborhood plus the next-nearest 16 cells. Shaded regions show 95% confidence intervals.
(TIFF)

Figure S2 **Change in the median distance (± SE for 100 realizations) between newly-established, randomly-placed** ***A. sericeasur*** **nests and the nearest existing nest in the previous year for two scenarios.** 1) if the number of nests is kept constant at the 2005 abundance (310 nests) while the number of available sites (shade trees) is reduced each year in accordance with the actual recorded pruning and 2) if the number of trees is kept constant at the 2005 level while the number of ant nests is changed each year according to the increasing abundance observed in the field. In scenario 1 (the black line in the figure), the distance between new nests and established nests increases due to the lower density of the available trees. In scenario 2 (the red line in the figure), the median distance decreases due to the increased overall density of ant nests in the plot. Both of these countervailing mechanisms were operative in the actual, observed field conditions.
(TIFF)
