# Supplementary figures and images for: Population Responses to Environmental Change in a Tropical Ant: The Interaction of Spatial and Temporal Dynamics
Source: PLoS One. 2014 May 19;9(5):e97809. doi: 10.1371/journal.pone.0097809 (PMC4026481; doi:10.1371/journal.pone.0097809)

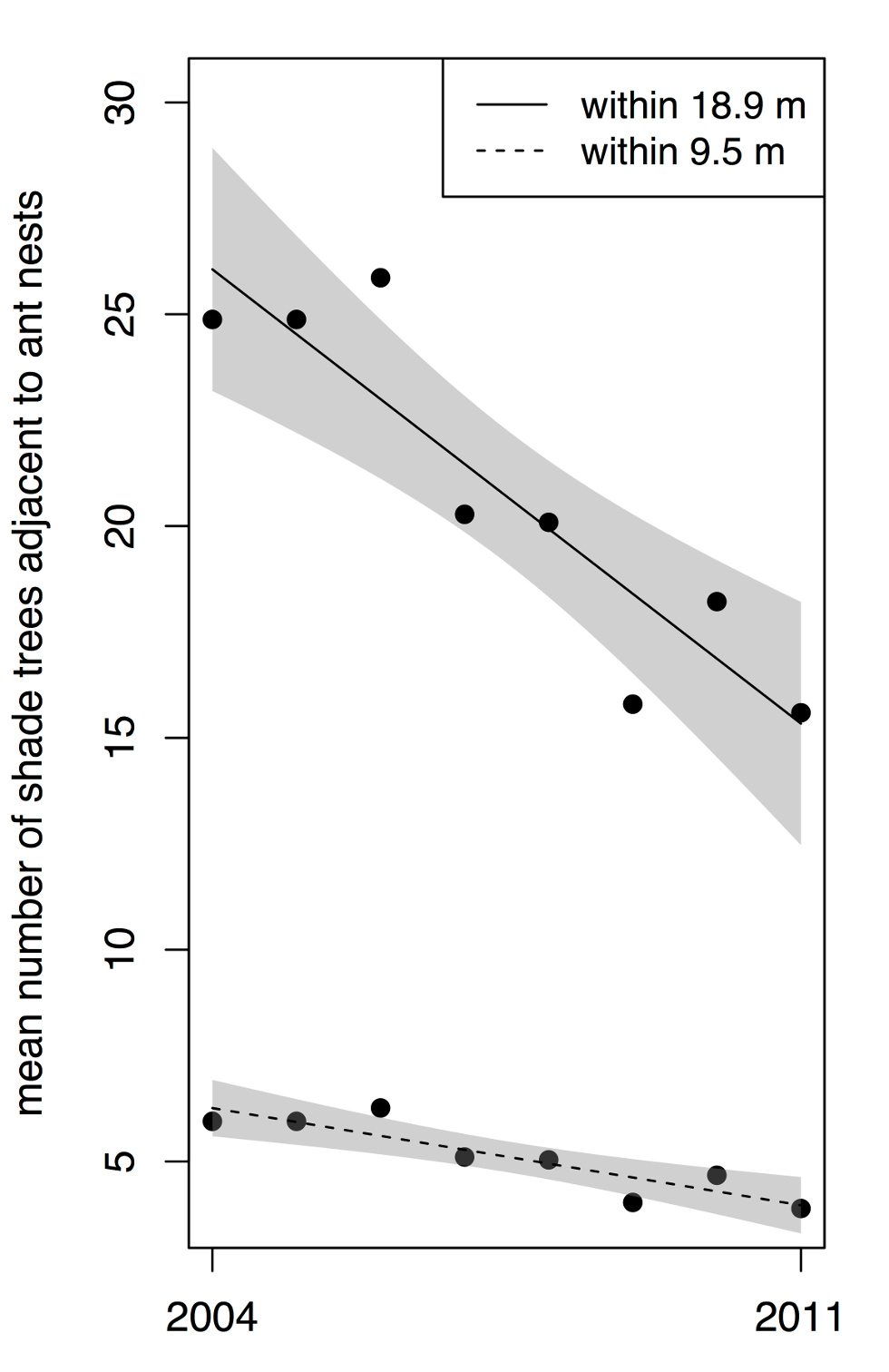

Supplement: Figure S1 — Mean number of shade trees adjacent to ant nests. Dashed line (R2 = 0.78, P<0.01) shows the mean number of shade trees within 9.5 m of ant nests, which corresponds to the eight nearest cells, i.e., the Moore neighborhood, in the cellular automata model. Solid line (R2 = 0.80, P<0.01) shows the mean number of shade trees within 18.9 m of the ant nests, which corresponds to the Moore neighborhood plus the next-nearest 16 cells. Shaded regions show 95% confidence intervals. (TIFF) [file pone.0097809.s001.tiff]

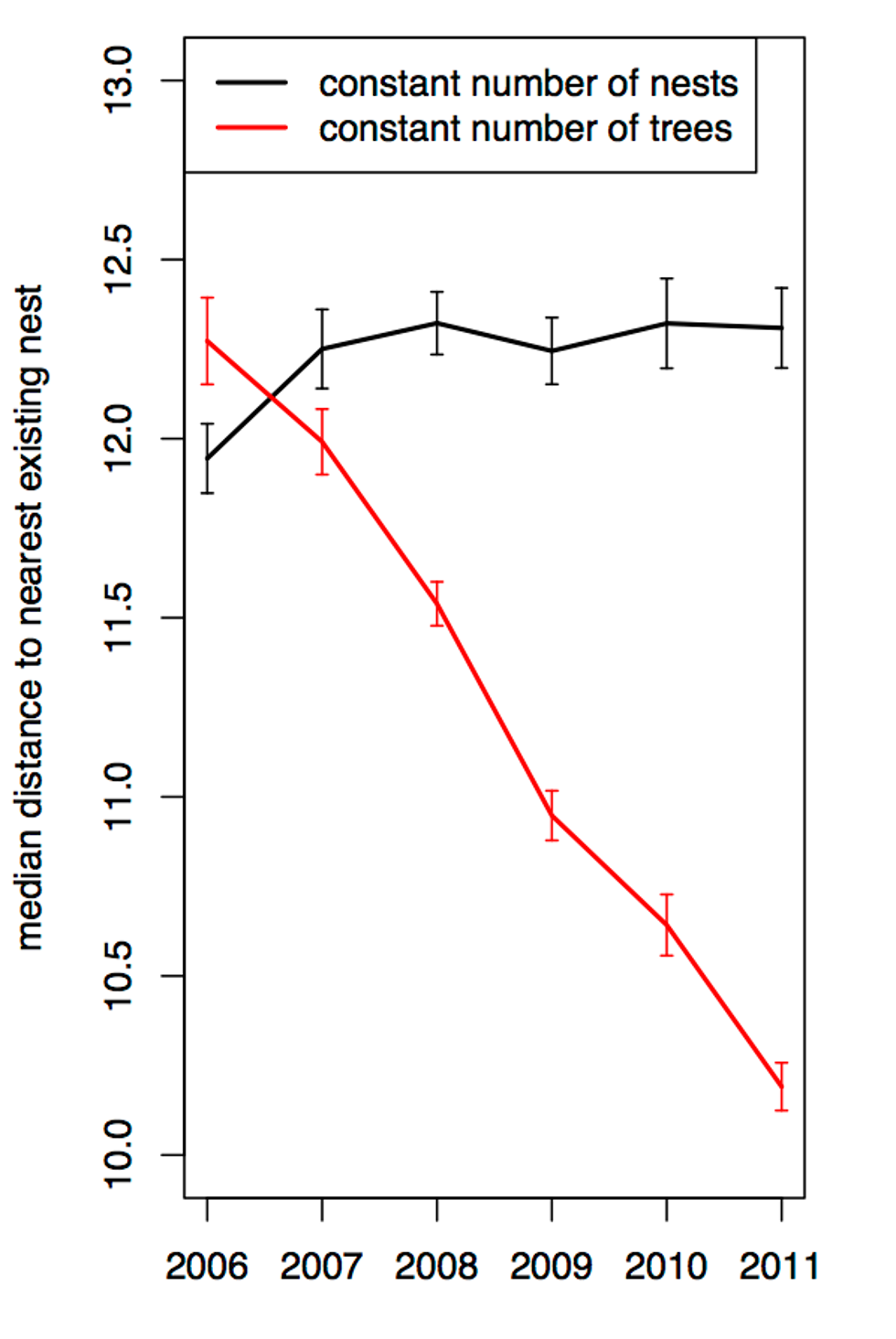

Supplement: Figure S2 — Change in the median distance (± SE for 100 realizations) between newly-established, randomly-placed A. sericeasur nests and the nearest existing nest in the previous year for two scenarios. 1) if the number of nests is kept constant at the 2005 abundance (310 nests) while the number of available sites (shade trees) is reduced each year in accordance with the actual recorded pruning and 2) if the number of trees is kept constant at the 2005 level while the number of ant nests is changed each year according to the increasing abundance observed in the field. In scenario 1 (the black line in the figure), the distance between new nests and established nests increases due to the lower density of the available trees. In scenario 2 (the red line in the figure), the median distance decreases due to the increased overall density of ant nests in the plot. Both of these countervailing mechanisms were operative in the actual, observed field conditions. (TIFF) [file pone.0097809.s002.tiff]
